# Poor Quality for Poor Women? Inequities in the Quality of Antenatal and Delivery Care in Kenya

**DOI:** 10.1371/journal.pone.0171236

**Published:** 2017-01-31

**Authors:** Jigyasa Sharma, Hannah H. Leslie, Francis Kundu, Margaret E. Kruk

**Affiliations:** 1 Department of Global Health and Population, Harvard T. H. Chan School of Public Health, Boston, MA, United States of America; 2 National Council for Population and Development, Nairobi, Kenya; Yale School of Public Health, UNITED STATES

## Abstract

**Background:**

Quality of healthcare is an important determinant of future progress in global health. However, the distributional aspects of quality of care have received inadequate attention. We assessed whether high quality maternal care is equitably distributed by (1) mapping the quality of maternal care in facilities located in poorer versus wealthier areas of Kenya; and (2) comparing the quality of maternal care available to Kenyans in and not in poverty.

**Methods:**

We assessed three measures of maternal care quality: facility infrastructure and clinical quality of antenatal care and delivery care, using indicators from the 2010 Kenya Service Provision Assessment (SPA), a standardized facility survey with direct observation of maternal care provision. We calculated poverty of the area served by antenatal or delivery care facilities using the Multidimensional Poverty Index. We used regression analyses and non-parametric tests to assess differences in maternal care quality in facilities located in more and less impoverished areas. We estimated effective coverage with a minimum standard of care for the full population and those in poverty.

**Results:**

A total of 564 facilities offering at least one maternal care service were included in this analysis. Quality of maternal care was low, particularly clinical quality of antenatal and delivery care, which averaged 0.52 and 0.58 out of 1 respectively, compared to 0.68 for structural inputs to care. Maternal healthcare quality varied by poverty level: at the facility level, all quality metrics were lowest for the most impoverished areas and increased significantly with greater wealth. Population access to a minimum standard (≥0.75 of 1.00) of quality maternal care was both low and inequitable: only 17% of all women and 8% of impoverished women had access to minimally adequate delivery care.

**Conclusion:**

The quality of maternal care is low in Kenya, and care available to the impoverished is significantly worse than that for the better off. To achieve the national targets of maternal and neonatal mortality reduction, policy initiatives need to tackle low quality of care, starting with high-poverty areas.

## Introduction

While the Millennium Development Goals (MDG) strategy was successful in expanding coverage of antenatal care (ANC) and skilled attendance at birth in low- and middle-income countries (LMICs), improvements in maternal and child health outcomes did not always follow.[1–3] In Kenya, for example, ANC coverage and presence of a skilled attendant at birth each increased twenty percentage points from 2000 to 2015, but maternal and neonatal mortality remain high (510 per 100,000 live births and 22.2 per 1,000 live births respectively, in 2015).[4, 5] One reason for this is shortcomings in the quality of health care available to women and children, an issue that is beginning to receive international attention.[6–13]

The inverse care law posits that the availability of good medical or social care varies inversely with the need of the population served.[14] While wealth disparities in mortality are well documented, less is known about the contribution of poor quality of care to these disparities. Prior research has documented inequities in reproductive and maternal health care access and outcomes within countries.[15] In Kenya, the burden of under-five mortality disproportionately affects rural, poorer and less-educated families.[16] Compared to children whose mothers have higher than secondary education, children whose mothers are not educated are 46% more likely to die before age five.[17] The poorest women received fewer essential services during ANC care and were four times as likely to deliver without a skilled attendant as women in the wealthiest quintile, according to the 2008–2009 Demographic and Health Survey (DHS).[16] A recent study indicates heterogeneity in quality of ANC across and within Kenyan provinces but did not detect statistically significant variation in ANC quality by women’s education level.[18] However, there are limited data on equity in maternal health care quality, particularly beyond ANC. Scarce health systems data, lack of systematically collected quality measures, and limited availability of timely subnational wealth data have constrained such inquiry in LMIC to date.

In this paper, we analyze inequities in the quality of maternal health care in Kenya. We use three measures of maternal care quality: an index of facility infrastructure for maternal care, and indexes of ANC clinical quality and delivery care clinical quality. We combine these measures with population data to (1) describe the geographic distribution of poverty and maternal care quality in Kenya, and (2) compare the quality of maternal care available to Kenyans in and not in poverty.

## Methods

### Study sample

We combined data from multiple sources for this analysis. We drew our sample of maternal care facilities in Kenya from the Service Provision Assessment (SPA) survey conducted by the DHS Program in 2010. SPA is a standardized, facility-based cross-sectional survey with direct observation of maternal care provision, designed to be representative of the health system (public and privately run facilities) at national and regional levels. We limited our sample of facilities to those providing at least one maternal care service, whether ANC or delivery care.

Demographic data were extracted from two sources: the Oxford Poverty and Human Development Initiative and the 2014 population-representative DHS.

The 2010 Constitution established 47 counties in Kenya. We obtained county boundaries from the GADM database version 2.8, a spatial database for global administrative areas, and the estimated 2010 population density from WorldPop (Creative Commons Attribution 4.0 International License).[19] For mapping purposes, we obtained the location of all health facilities in Kenya from Kenya Open Data. Analysis proceeded in two stages: description of poverty and maternal care quality at the county level and assessment of poverty as a predictor of facility quality based on facility catchment areas.

### Poverty

We defined poverty using the multidimensional poverty index (MPI). The MPI measures poverty using ten indicators in three dimensions: education (years of schooling and school attendance), health (child mortality and nutrition) and standard of living (cooking fuel, sanitation, water, electricity, floor and asset ownership). The MPI reflects both the incidence and the average intensity of poverty. A person is identified as impoverished if she is deprived in at least one third of the weighted indicators. Using data from the 2008–2009 DHS, the Oxford Poverty and Human Development Initiative estimated MPI per square kilometer (km) for the full country, with accompanying uncertainty estimates based on width of the 95% credible interval.[20] We calculated number of those impoverished, average poverty and average uncertainty per county. Based on data showing that nearly 90% of women in Kenya lived within 5 km of a health facility,[21] we defined facility catchment area using a buffer zone with a 5-km radius and calculated average proportion of individuals in poverty within these areas. We classified counties and catchment areas into five levels (poorest to richest) using even intervals of 20% poverty.

As a secondary metric, we extracted household wealth index from the 2014 DHS survey and calculated average wealth per county (weighted with household sampling weights). The 2014 DHS was designed to provide representative summaries at the county level. We classified counties into wealth quintiles based on the thresholds applied in defining wealth quintiles for households.

### Maternal care quality

The Institute of Medicine’s canonical report on quality of healthcare proposes that a high quality health system is safe, effective, patient centered, timely, efficient, and equitable.[22] We operationalized these measures using Donabedian’s framework of structure, process and outcome.[23] Structural elements, such as availability of medicines and equipment, represent necessary, but not sufficient, conditions for the delivery of a given quality of health care. Process indicators represent the closest approximation of the actual quality of health care offered, as these include both technical (appropriate delivery of clinical procedures) and interpersonal (client-provider interactions) aspects of healthcare delivery. Outcome indicators measure health improvements attributable to medical care, for example, under-five mortality rate. These measures, however, may be affected by factors other than quality of healthcare delivered that influence outcomes.

Using Donabedian’s framework, we identified indicators for structural inputs to maternal care (infrastructure, staffing, and equipment) as well as clinical care processes in antenatal care and delivery care to cover the spectrum of maternal care. Indicators for maternal care structure were constructed using data extracted from the SPA facility audit and provider interviews. This index included thirty items covering infrastructure (e.g., functional water and electricity), staffing (e.g., availability of 24-hour delivery care, staff training in ANC or delivery care), and equipment and supplies, such as iron folate for ANC and injectable uterotonics for delivery. See S1 Fig for details. Facilities offering only one of ANC or delivery care were scored based on general facility infrastructure items and those elements relevant to the service provided.

Clinical care processes were assessed using direct observation of ANC and deliveries in a subset of facilities. We created an ANC clinical quality index using forty actions providers are expected to perform during all first ANC visits, drawn from the Focused Antenatal Care Model Checklist;[24] sample items include assessment of client history, counseling on danger signs, and administration of tetanus toxoid injection and HIV test (S2 Fig). To measure clinical quality of delivery care, we applied the quality of the process of intrapartum and immediate postpartum care (QoPIIPC) metric validated by Tripathi et al.[25] to the SPA data, extracting indicators matching eighteen of the twenty QoPIIPC items. These included checking woman’s blood pressure, washing hands before any examination, and timely administration of uterotonic (S3 Fig). Clinical observations were averaged within each facility, weighted with the patient sampling weight rescaled within facility. For each quality index, indicators were averaged to provide a facility summary score from 0 to 1, with missing values excluded. Completeness of each indicator is shown in S1 Table; missingness was minimal for infrastructure and ANC observations, but substantial (up to 32%) for observed deliveries, particularly for items at early stages of labor that frequently occur outside of the health facility.

### Covariates

We calculated patient load per facility based on reported delivery clients (including cesarean section as applicable) and reported ANC visits in the past 12 months.

### Statistical analysis

We first described quality of care and population in poverty at the county level. We calculated weighted averages of each quality metric per county, weighting facilities by total maternal care visits (deliveries plus ANC visits), total ANC visits, and total deliveries for the maternal infrastructure, ANC quality, and delivery quality indices respectively. We calculated the weighted standard error (SE) of each quality index within county, using the full sample standard deviation (SD) for counties with a single facility, and generated a 95% confidence interval (CI) for each county using this SE. We present summary statistics of county population and health facility access by strata of poverty level. We calculated average quality by level of poverty and used average SE by poverty level to generate 95% CIs for each estimate. A non-parametric test for trend was employed to assess differences in maternal care quality by county poverty level.

To quantify population access to quality care, we defined a threshold of at least 0.75 on each index as minimally adequate maternal care quality. Given the lack of universally defined minimum quality standards, we selected this threshold on the premise that women should receive most of these basic items at minimum. We calculated access to adequate maternal care by summing the total population and impoverished individuals within counties with adequate care quality. We repeated this analysis using the bounds of the 95% CI for county quality estimates to quantify uncertainty around population access to quality care.

In order to obtain a more precise understanding of quality of care accessible to poor women, we assessed poverty and quality at the facility level; we regressed each quality index on poverty level of the surrounding area, clustered by county to account for adjacent facilities with overlapping catchment areas. We used the poorest areas (>80% poverty) as the reference group for all models except for quality of delivery care: due to the small number of facilities with directly observed deliveries in the poorest quintile (N = 2), we collapsed the two poorest quintiles as the reference category for these models.

We conducted multiple sensitivity analyses. To assess sensitivity to the definition of the catchment area, we repeated the analysis redefining the catchment area for hospitals to cover 20 km. To test robustness to uncertainty in the MPI estimates, we excluded facilities with average uncertainty greater than 50%. To assess the reproducibility of the results with an alternative poverty metric, we regressed average quality per county on wealth quintile as measured in the 2014 DHS.

Statistical analysis was done using Stata 14.1 (StataCorp, Texas) and mapping using QGIS Version 2.12 (Free Software Foundation, Massachusetts) and ArcGIS 10.3.1 (Esri, California).

### Ethical approval

The original survey implementers obtained ethical approvals for data collection; the Harvard University Human Research Protection Program deemed this secondary analysis exempt from human subjects review.

## Results

**Fig 1:**
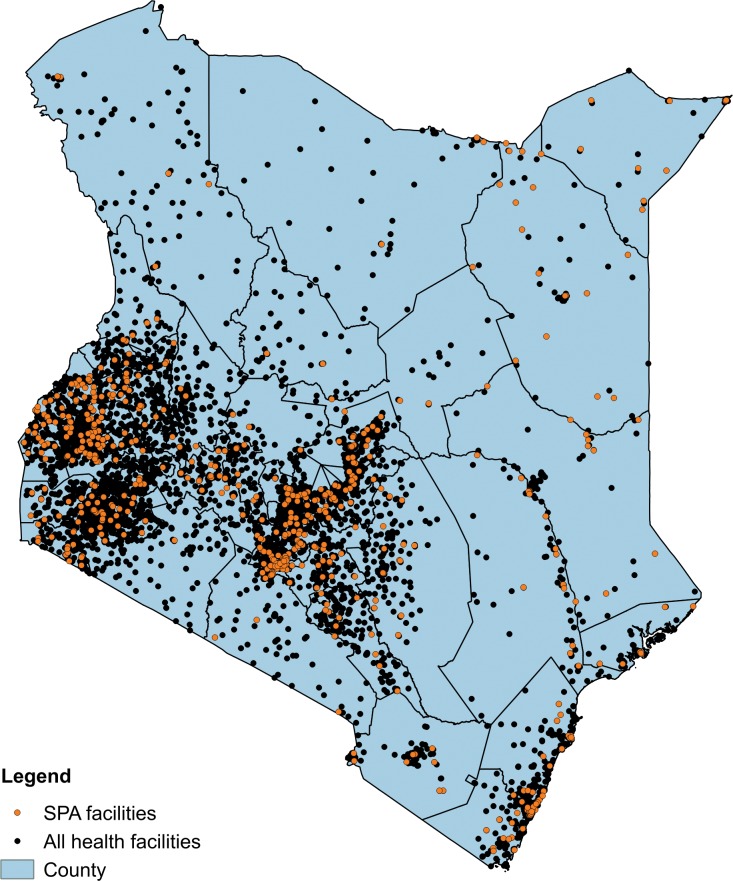
Kenyan health system and facilities sampled in SPA. Location of all health facilities in Kenya obtained from Kenya Open Data based on data from the Kenya National Bureau of Statistics in 2007.

The 2010 SPA successfully assessed 695 of 703 sampled facilities (98.9% response rate); 568 of these facilities provided at least one maternity care service. Four facilities (0.7%) lacked valid geographic data, yielding an analytic sample of 564 facilities with maternal care services (557 offering ANC and 400 providing normal delivery services). As shown in Fig 1, the geographic distribution of sampled facilities followed the distribution of the health system as a whole, with most facilities located in the densely populated areas of Nairobi, the southern coast, and south western Kenya. Forty five percent of the population of Kenya (18.1 million individuals) lived within 5 km of a sampled facility (S4 Fig shows population density and catchment areas). An average of 12 facilities providing maternal care were assessed per county (range 2–52) with a total of 544 observations of first ANC visits and 621 labor and delivery observations, although one and five of the 47 counties lacked data on directly observed antenatal care and delivery care, respectively. These counties were significantly more likely to be poor than counties with observations (Fisher’s exact test p<0.05).

Table 1 shows the distribution of counties and health facilities by poverty level; a plurality of Kenyans lived in counties with 40–60% of the population in poverty. Nairobi is the only county with <20% poverty in Kenya. The distribution of health facilities largely matched population share, with the exception of a greater concentration of cesarean section-capable facilities in wealthier counties.

**Table 1:** Distribution of population and health services in Kenya by poverty.

	Counties	Population (thousands)	Facilities assessed	Facility services		
				ANC	Delivery	C-section
Poverty level	N (%)	N (%)	N (%)	N (%)	N (%)	N (%)
80%+	6 (12.8)	2,189.7 (5.4)	52 (7.5)	41 (7.4)	28 (7.0)	5 (3.4)
60–80%	10 (21.3)	6,256.1 (15.5)	114 (16.5)	93 (16.7)	75 (18.8)	23 (15.4)
40–60%	21 (44.7)	18,239.2 (45.1)	271 (39.3)	241 (43.3)	177 (44.3)	55 (36.9)
20–40%	9 (19.1)	10,084.8 (24.9)	165 (23.9)	130 (23.3)	89 (22.3)	46 (30.9)
0–20%	1 (2.1)	3,690.6 (9.1)	88 (12.8)	52 (9.3)	31 (7.8)	20 (13.4)
Total	47	40,460.4	690	557	400	149

SPA surveyed 695 facilities; 5 could not be matched to county due to lack of detailed geographic data. 564 of these 690 facilities provided at least one maternal care service

**Fig 2:**
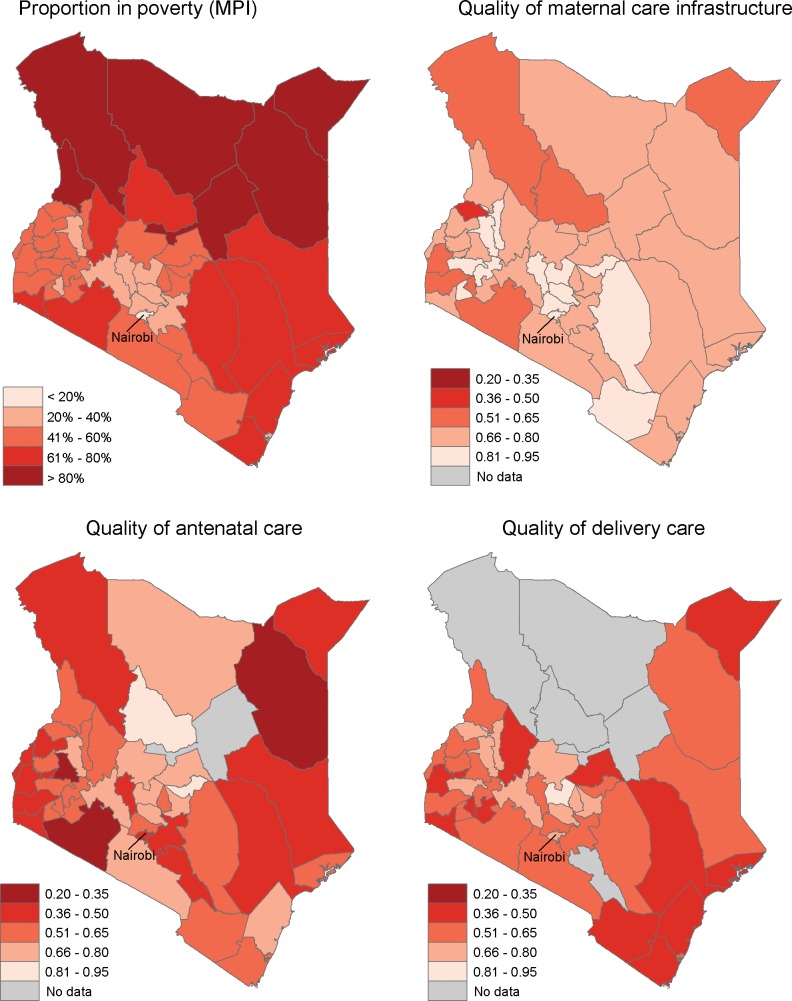
Geographic distribution of poverty and quality maternal care.

**Fig 3:**
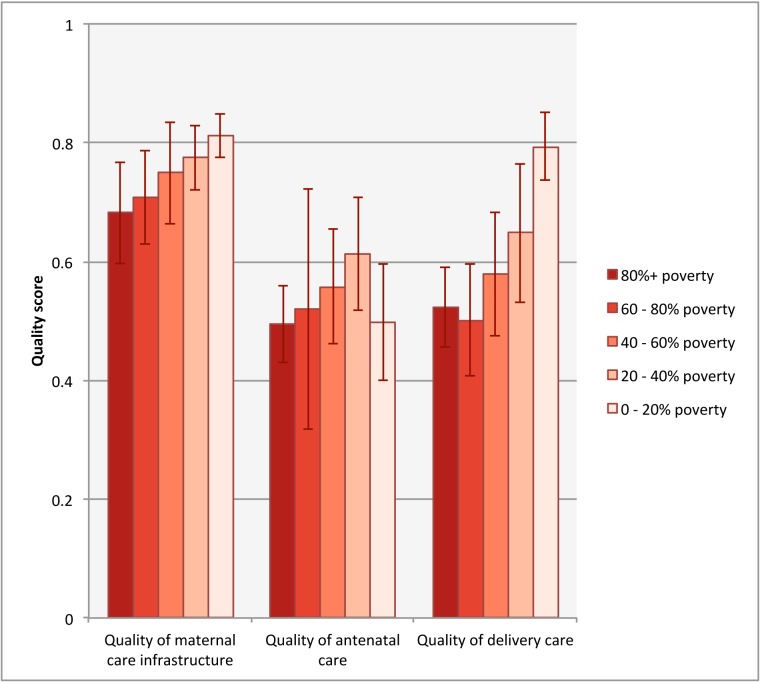
Quality of maternal health services by county poverty level. Non-parametric test for trend significant for quality of maternal care infrastructure and quality of delivery care, p<0.05.

Quality of maternal care services was low in assessed facilities, with average scores of 0.68 (SD 0.15) for maternal care infrastructure, 0.52 (SD 0.19) for ANC quality, and 0.58 (SD 0.17) for delivery quality. As shown in Fig 2, quality of maternal care infrastructure was more uniform across the country as well as being higher than quality of ANC or delivery care. Considerable heterogeneity is apparent between quality on the three domains in a given county, suggesting uneven quality throughout the course of pregnancy and delivery. Visual inspection suggests higher quality for infrastructure and delivery care in the wealthiest counties around Nairobi. Fig 3, which pools data from across the country, shows that quality of care increases with decreasing poverty across the three quality domains. This trend was significant for both maternal care infrastructure and delivery care (non-parametric test for trend p<0.05).

We also found inequities in population access to minimally adequate maternal care (quality at least 0.75 out of 1.00). Fifty-six percent of the population in poverty lived in counties with adequate maternal care infrastructure compared to 69% of those not in poverty (63% of total population, uncertainty interval 29% to 80%; see S2 Table). Adequate delivery care quality at the county level was available to 8% of impoverished women and 24% of those not impoverished (17% in total, uncertainty interval 3% to 41%). Access to quality ANC was extremely low regardless of poverty: 9% of the population lived in counties with minimally adequate quality (uncertainty interval 3% to 30%).

Results of models that regressed facility quality on level of poverty in surrounding areas (Table 2) show that quality scores increased steadily with decreasing poverty, reaching estimated differences of 16% on infrastructure for maternal care quality (95% CI 12%, 20%), 19% on ANC quality (7%, 31%), and 12% on delivery care quality (95% CI 0%, 25%) between the richest and poorest or, for delivery care, poorest and second poorest areas (see S3 Table for distribution of facilities and observations by poverty). Women living in intensely impoverished areas could expect to receive only one third of basic clinical actions in their first ANC visit and half of basic essential evidence-based actions in delivery care compared to approximately 60% in each area for women in wealthier (<60% poverty) areas. These findings were largely robust to sensitivity checks for the definition of catchment areas, uncertainty in poverty estimation, and verification using an alternate wealth measure, although differences in ANC quality did not reach significance in the county-level analysis using DHS wealth quintiles (see S4 Table for sensitivity results).

**Table 2:** Association between poverty and maternal care quality.

	Quality of maternal care infrastructure	Clinical quality of antenatal care	Clinical quality of delivery care
Main analysis: facilities with 5 km catchment areas	β (95% CI)	β (95% CI)	β (95% CI)
	N = 564	N = 285	N = 169
Poverty level			
80%+	0.00 (REF)	0.00 (REF)	0.00 (REF)
60–80%	**0.05 (0.01, 0.09)**	**0.14 (0.00, 0.27)**	
40–60%	**0.06 (0.03, 0.09)**	**0.14 (0.01, 0.27)**	0.00 (-0.05, 0.06)
20–40%	**0.12 (0.08, 0.16)**	**0.27 (0.13, 0.40)**	**0.10 (0.02, 0.18)**
0–20%	**0.16 (0.12, 0.20)**	**0.19 (0.07, 0.31)**	0.12 (0.00, 0.25)
Intercept	0.59 (0.57, 0.61)	0.34 (0.22, 0.46)	0.52 (0.49, 0.56)

All models clustered by county to account for correlation between catchment areas of neighboring facilities

The analysis for clinical quality of delivery care uses facilities in areas with 60%+ poverty as the reference category due to the small number of facilities in the poorest group.

Results in bold are significant at p≤0.05.

## Discussion

In this paper, we described the quality of maternal care in Kenya across three dimensions: facility infrastructure and clinical process quality of antenatal and delivery care. Our findings indicate low overall quality, particularly for clinical quality of antenatal and delivery care. We also found substantial heterogeneity in quality by population wealth: all quality indicators were lowest for the poorest population quintile and increased with increasing wealth. Evidence indicates similar inequities in population access to a minimal standard of care as well. This is consistent with evidence from other studies that have noted a quality deficit in health services available to the poor.[26–30]

Few studies have addressed inequities in quality of maternal care. A study in five African countries, including Kenya, found significantly lower quality of basic maternal care functions (infrastructure and signal functions) in public facilities compared to private;[13] the location of private facilities in wealthier areas may be one mechanism underlying inequities in access to quality care. A study in Mexico identified lower coverage of adequate (timely, repeated, and thorough) ANC among women with less education and lower SES.[31] However, in a recent study in Kenya, Lee et al. documented substantial variation in quality of ANC across and within provinces but found no statistically significant association with women’s education level.[18] In contrast, we find significantly poorer quality for women in poor areas. Our findings pertain to the quality of care accessible to women in more or less impoverished areas rather than to care received by a specific individual; we also employed more comprehensive metrics of socioeconomic disadvantage and maternal care quality. We found stronger evidence for inequities in access for maternal care infrastructure and quality of delivery care than ANC, underscoring the importance of assessing quality of care throughout pregnancy and delivery.

Infrastructure for maternal care scored higher than clinical care quality and varied the least across facilities and counties. This suggests partial fulfillment of the 1994–2010 Kenya Health Policy Framework goal of ensuring equitable allocation of resources to reduce health disparities.[32] However, we found considerable wealth gaps in quality for delivery care: while 24% of poor women had access to minimally adequate quality of delivery care, only 8% of poor women had access to such care. Evidence from multiple LMICs indicates that provider effort is a more consistent determinant of primary care quality than structural constraints;[33] as such, assessment of resources and services alone would substantially understate the disparities in access to high quality care for poor women. Deficits in care delivery relative to infrastructure are not unexpected: interventions to improve clinical quality are often more challenging to implement than provision of supplies. Human capacity interventions have been linked to modest improvements at best;[34, 35] care as delivered often falls short of provider’s own knowledge, let alone evidence-based guidelines.[33]

Researchers estimate that competent intrapartum care could avert one third of maternal deaths and over half of neonatal deaths annually;[36] a recent study suggested most avertable neonatal mortality in Malawi could be associated with low-quality delivery facilities.[37] Poor quality delivery care for poor women may thus explain some of the disparities in maternal and newborn survival between rich and poor women, which are often concealed by national averages.[38] The five poorest counties in Kenya by MPI experience the highest maternal mortality rates in the country, ranging from 790 to 3,795 per 100,000 live births, far above the national average of 495 per 100,000.[39] Achieving the national goal of reducing maternal mortality to 113 per 100,000 by 2030[32] will require intensive intervention in these most impoverished areas.

Our study also shows that access to a basic threshold of quality maternal care services is low in Kenya: minimally adequate quality of delivery care, at county level, was available only to 17% of the population, and effective ANC coverage to 9%. Our findings are similar to those reporting effective coverage from other sub-Saharan Africa countries. Delivery facilities in Malawi provided on average only 16 of 25 essential items for quality maternal care in 2013.[37] A study from Ghana indicates that 39% of the facilities provided substandard routine delivery care, and 63% provided poor emergency obstetric care, resulting in 50% of births in the study area not receiving high quality care despite a 68% coverage of facility-based births.[40] Likewise, a study of ANC quality in Zambia indicated that only 3% of the facilities provided optimal ANC service, while 47% of facilities provided adequate service, and the remaining 50% offered inadequate service.[41] Collectively, these findings lend credence to suggestions that ineffective health care coverage is common in sub-Saharan Africa and a likely explanation for persistent poor health outcomes despite increased access to care.

There are several limitations in this analysis. First, we are not able to link facilities to specific patient populations that use them, instead drawing catchments around facilities. Catchment areas around private facilities in particular may not reflect the actual users of the facility. While bypassing of facilities may bias our results, studies show that the poor tend to bypass less than wealthier women, limiting this threat to our inference.[42] Second, the MPI estimates are based on interpolations subject to uncertainty, although our results were unchanged limiting to areas with higher certainty. Third, missing data and small number of observations limits analysis of the quality of delivery care metric. Although direct observation is the gold standard of measurement, it is difficult to obtain multiple, complete observations of deliveries, particularly for low-volume facilities most common in poor, sparsely populated areas. Incomplete information in assessing quality of delivery care could bias the results, although this measurement error is more likely to attenuate effect estimates than overstate them. Finally, the SPA sample is not constructed to be representative at the county level or catchment area. County-level estimates should be interpreted with the conservative uncertainty estimates provided. Generalizing the facility-level analysis to the full country assumes that the population surrounding these facilities is representative of the population around all health facilities, at least in terms of socioeconomic status. This assumption is plausible, particularly given that sampled areas covered 45% of the population.

To our knowledge, this is the first study to use nationally-representative data to quantify quality of antenatal and delivery care using the gold-standard measure of direct observation of over 500 ANC visits and over 600 deliveries. We have combined these facility data with population metrics to provide greater insight into inequities in access to effective coverage of maternal health services throughout pregnancy and delivery. Our results are robust to several sensitivity checks.

Our findings offer support for the inverse care law for maternal health care in Kenya. We have demonstrated substantial deficiencies in quality, as well as a steep wealth gradient in access to quality maternity care. This work has several policy implications for maternal care in Kenya. The 2014–2030 Health Policy Framework includes equitable distribution of health services and interventions as a policy principle.[32] Basic maternal care is broadly accessible to Kenyan women, and the policy of free delivery care in public hospitals has led to large increases in coverage of skilled attendance at births since 2000.[4] However, this focus on access to care will be insufficient to reduce avertable morbidity and mortality among the most vulnerable women and children if the quality of health services available to them is poor. To achieve the country’s ambitious targets of a 59% reduction in neonatal mortality and a 77% reduction in maternal mortality by 2030, improving the quality of maternal care must become a health system policy priority in Kenya. Improved care for poor women should be at the forefront of such efforts to correct wealth disparities and to avert the most maternal and newborn deaths.

In Kenya and in other high mortality settings, this evidence should spur action to measure quality of care beyond availability of services and supplies, to track progress in quality improvement, and to collect wealth-disaggregated data on quality and effective coverage. Quality improvement initiatives need to prioritize improving care in clinics serving poor communities before others. Further research is also warranted to better understand the causes of inequity in quality of facility-based maternal care. Coverage with poor quality of care for the most vulnerable does not represent progress for women and newborns.

## Supporting Information

S1 Fig Quality of maternal care infrastructure. (GIF)

S2 Fig Quality of clinical care in first ANC visits (N = 544 observations). *Excluded from quality metric for primiparous women.
(GIF)

S3 Fig Quality of processes of intrapartum and immediate postpartum care (N = 621 observations). Notes: One item from the validated index, HIV testing, was not assessed in the Kenya SPA; two other items from the validated index, on heavy bleeding or headaches experienced during pregnancy, were asked as a single item in the SPA; there are thus 18 items on the metric calculated here.
(GIF)

S4 Fig Areas surrounding SPA sample of maternal health (5 kilometer radius around facilities) and population density in Kenya, with inset of Nairobi. (TIF)

S1 Table Completeness of indicators for facilities and observations. (DOCX)

S2 Table Population access to minimally adequate standard of care. (DOCX)

S3 Table Distribution of facilities and observations by poverty of surrounding area. (DOCX)

S4 Table Robustness checks of association between poverty and maternal care quality. (DOCX)

## Abbreviations

ANC: Antenatal care
CI: Confidence interval
DHS: Demographic and Health Survey
km: Kilometer
LMIC: low- and middle-income countries
MDG: Millennium Development Goals
MPI: Multidimensional poverty index
QoPIIPC: Quality of the process of intrapartum and immediate postpartum care
SD: Standard deviation
SE: Standard error
SPA: Service Provision Assessment
