# Supplementary figures and images for: Poor Quality for Poor Women? Inequities in the Quality of Antenatal and Delivery Care in Kenya
Source: PLoS One. 2017 Jan 31;12(1):e0171236. doi: 10.1371/journal.pone.0171236 (PMC5283741; doi:10.1371/journal.pone.0171236)

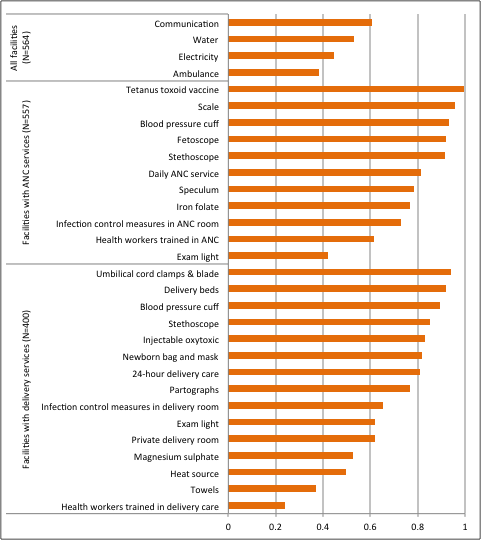

Supplement: S1 Fig — (GIF) [file pone.0171236.s001.gif]

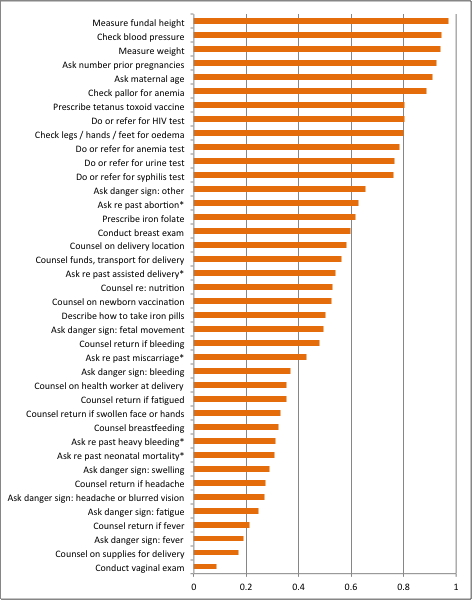

Supplement: S2 Fig — *Excluded from quality metric for primiparous women. (GIF) [file pone.0171236.s002.gif]

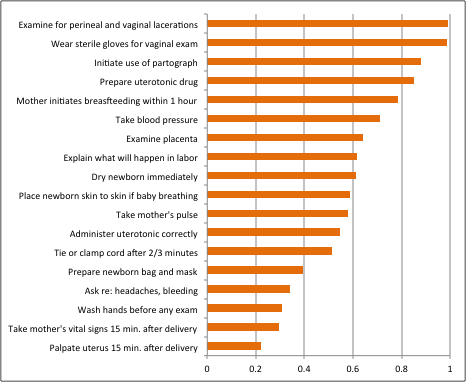

Supplement: S3 Fig — Notes: One item from the validated index, HIV testing, was not assessed in the Kenya SPA; two other items from the validated index, on heavy bleeding or headaches experienced during pregnancy, were asked as a single item in the SPA; there are thus 18 items on the metric calculated here. (GIF) [file pone.0171236.s003.gif]

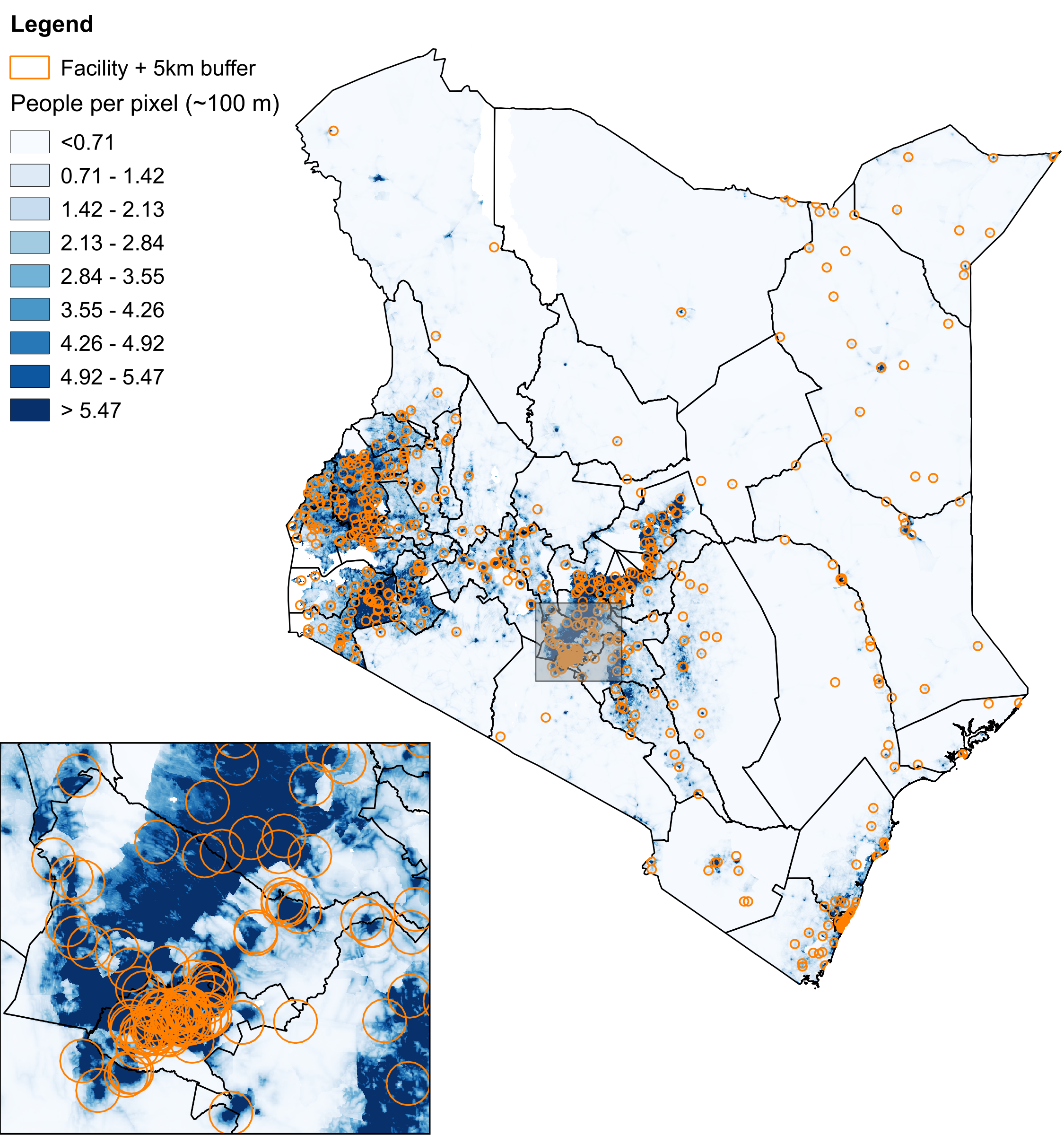

Supplement: S4 Fig — (TIF) [file pone.0171236.s004.tif]
