# Supplementary material for: Poor Quality for Poor Women? Inequities in the Quality of Antenatal and Delivery Care in Kenya
Source: PLoS One. 2017 Jan 31;12(1):e0171236. doi: 10.1371/journal.pone.0171236 (PMC5283741; doi:10.1371/journal.pone.0171236)
Supplement: S1 Table — (DOCX) [file pone.0171236.s005.docx]

S1 Table: Completeness of indicators for facilities and observations

|  | % Complete | % Missing |
| --- | --- | --- |
| **Infrastructure (N=564 facilities, 557 with ANC and 400 with delivery care)** | | |
| Communication | 99.8% | 0.2% |
| Water | 99.5% | 0.5% |
| Electricity | 100.0% | 0.0% |
| Ambulance | 100.0% | 0.0% |
| Tetanus toxoid vaccine | 88.7% | 11.3% |
| Scale | 99.8% | 0.2% |
| Blood pressure cuff | 100.0% | 0.0% |
| Fetoscope | 99.8% | 0.2% |
| Stethoscope | 100.0% | 0.0% |
| Daily ANC service | 100.0% | 0.0% |
| Speculum | 99.8% | 0.2% |
| Iron folate | 99.5% | 0.5% |
| Infection control measures in ANC room | 100.0% | 0.0% |
| Health workers trained in ANC | 98.0% | 2.0% |
| Exam light | 100.0% | 0.0% |
| Umbilical cord clamps & blade | 100.0% | 0.0% |
| Delivery beds | 99.5% | 0.5% |
| Blood pressure cuff | 96.8% | 3.3% |
| Stethoscope | 96.8% | 3.3% |
| Injectable oxytoxic | 100.0% | 0.0% |
| Newborn bag and mask | 100.0% | 0.0% |
| 24-hour delivery care | 93.8% | 6.3% |
| Partographs | 98.8% | 1.3% |
| Infection control measures in delivery room | 100.0% | 0.0% |
| Exam light | 96.8% | 3.3% |
| Private delivery room | 100.0% | 0.0% |
| Magnesium sulphate | 100.0% | 0.0% |
| Heat source | 100.0% | 0.0% |
| Towels | 100.0% | 0.0% |
| Health workers trained in delivery care | 99.5% | 0.5% |
| **ANC quality (N=544 observations)** |  |  |
| Measure fundal height | 99.8% | 0.2% |
| Check blood pressure | 99.8% | 0.2% |
| Measure weight | 99.8% | 0.2% |
| Ask number prior pregnancies | 99.8% | 0.2% |
| Ask maternal age | 100.0% | 0.0% |
| Check pallor for anemia | 99.8% | 0.2% |
| Prescribe tetanus toxoid vaccine | 99.8% | 0.2% |
| Do or refer for HIV test | 99.6% | 0.4% |
| Check legs / hands / feet for oedema | 99.8% | 0.2% |
| Do or refer for anemia test | 99.6% | 0.4% |
| Do or refer for urine test | 99.4% | 0.6% |
| Do or refer for syphilis test | 99.4% | 0.6% |
| Ask danger sign: other | 99.8% | 0.2% |
| Ask re past abortion* | 100.0% | 0.0% |
| Prescribe iron folate | 99.8% | 0.2% |
| Conduct breast exam | 99.4% | 0.6% |
| Counsel on delivery location | 99.8% | 0.2% |
| Counsel funds, transport for delivery | 100.0% | 0.0% |
| Ask re past assisted delivery* | 100.0% | 0.0% |
| Counsel re: nutrition | 99.8% | 0.2% |
| Counsel on newborn vaccination | 100.0% | 0.0% |
| Describe how to take iron pills | 100.0% | 0.0% |
| Ask danger sign: fetal movement | 99.4% | 0.6% |
| Counsel return if bleeding | 100.0% | 0.0% |
| Ask re past miscarriage* | 99.8% | 0.2% |
| Ask danger sign: bleeding | 99.8% | 0.2% |
| Counsel on health worker at delivery | 100.0% | 0.0% |
| Counsel return if fatigued | 100.0% | 0.0% |
| Counsel return if swollen face or hands | 100.0% | 0.0% |
| Counsel breastfeeding | 100.0% | 0.0% |
| Ask re past heavy bleeding* | 99.8% | 0.2% |
| Ask re past neonatal mortality* | 100.0% | 0.0% |
| Ask danger sign: swelling | 99.6% | 0.4% |
| Counsel return if headache | 99.8% | 0.2% |
| Ask danger sign: headache or blurred vision | 99.6% | 0.4% |
| Ask danger sign: fatigue | 99.4% | 0.6% |
| Counsel return if fever | 100.0% | 0.0% |
| Ask danger sign: fever | 99.4% | 0.6% |
| Counsel on supplies for delivery | 100.0% | 0.0% |
| Conduct vaginal exam | 98.7% | 1.3% |
| **Delivery quality (N=621 observations)** | |  |
| Examine for perineal and vaginal lacerations | 88.6% | 11.4% |
| Wear sterile gloves for vaginal exam | 89.7% | 10.3% |
| Initiate use of partograph | 80.0% | 20.0% |
| Prepare uterotonic drug | 91.0% | 9.0% |
| Mother initiates breasfteeding within 1 hour | 87.0% | 13.0% |
| Take blood pressure | 72.3% | 27.7% |
| Examine placenta | 88.9% | 11.1% |
| Explain what will happen in labor | 88.2% | 11.8% |
| Dry newborn immediately | 89.0% | 11.0% |
| Place newborn skin to skin if baby breathing | 88.2% | 11.8% |
| Take mother's pulse | 72.3% | 27.7% |
| Administer uterotonic correctly | 100.0% | 0.0% |
| Tie or clamp cord after 2/3 minutes | 88.7% | 11.3% |
| Prepare newborn bag and mask | 85.8% | 14.2% |
| Ask re: headaches, bleeding | 72.1% | 27.9% |
| Wash hands before any exam | 68.0% | 32.0% |
| Take mother's vital signs 15 min. after delivery | 87.4% | 12.6% |
| Palpate uterus 15 min. after delivery | 87.1% | 12.9% |
