# Supplementary material for: Poor Quality for Poor Women? Inequities in the Quality of Antenatal and Delivery Care in Kenya
Source: PLoS One. 2017 Jan 31;12(1):e0171236. doi: 10.1371/journal.pone.0171236 (PMC5283741; doi:10.1371/journal.pone.0171236)
Supplement: S2 Table — (DOCX) [file pone.0171236.s006.docx]

S2 Table: Percent of population with access to minimally adequate standard of care (quality at least 0.75 out of 1.00)

|  | Population percentage [95% Uncertainty Interval] | | |
| --- | --- | --- | --- |
|  | Total | Impoverished | Not impoverished |
| Quality of maternal care infrastructure | 63.0% [28.7, 79.5] | 56.3% [17.7, 75.4] | 68.8% [38.1, 83.1] |
| Quality of antenatal care | 8.9% [3.4, 30.9] | 10.2% [3.3, 32.1] | 7.8% [3.5, 30.0] |
| Quality of delivery care | 16.9% [2.9, 41.1] | 8.0% [2.3, 28.6] | 24.1% [3.4, 50.9] |
