# Supplementary material for: Poor Quality for Poor Women? Inequities in the Quality of Antenatal and Delivery Care in Kenya
Source: PLoS One. 2017 Jan 31;12(1):e0171236. doi: 10.1371/journal.pone.0171236 (PMC5283741; doi:10.1371/journal.pone.0171236)
Supplement: S3 Table — (DOCX) [file pone.0171236.s007.docx]

S3 Table: Distribution of facilities and observations by poverty of surrounding area

|  |  | ANC | | | Labor & Delivery | | |
| --- | --- | --- | --- | --- | --- | --- | --- |
| Poverty level | Maternal care facilities | Facilities with service | Facilities with observation | Total observations | Facilities with service | Facilities with observation | Total observations |
| 80%+ | 24 | 24 | 6 | 10 | 14 | 2 | 2 |
| 60 – 80% | 56 | 53 | 29 | 49 | 44 | 17 | 51 |
| 40 – 60% | 212 | 211 | 127 | 254 | 159 | 67 | 239 |
| 20 – 40% | 183 | 181 | 86 | 161 | 121 | 56 | 238 |
| 0 – 20% | 89 | 88 | 37 | 70 | 62 | 27 | 91 |
| Total | 564 | 557 | 285 | 544 | 400 | 169 | 621 |
