# Supplementary material for: Poor Quality for Poor Women? Inequities in the Quality of Antenatal and Delivery Care in Kenya
Source: PLoS One. 2017 Jan 31;12(1):e0171236. doi: 10.1371/journal.pone.0171236 (PMC5283741; doi:10.1371/journal.pone.0171236)
Supplement: S4 Table — (DOCX) [file pone.0171236.s008.docx]

S4 Table: Robustness checks of association between poverty and maternal care quality

|  | Quality of maternal care infrastructure | Clinical quality of antenatal care | Clinical quality of delivery care |
| --- | --- | --- | --- |
| **1: facilities with 5 / 20 km catchment areas** | β (95% CI) | β (95% CI) | β (95% CI) |
|  | N=564 | N=285 | N=169 |
| Poverty level |  |  |  |
| 80%+ | 0.00 (REF) | 0.00 (REF) | 0.00 (REF) |
| 60 - 80% | 0.01 (-0.05, 0.07) | **0.11 (0.01, 0.22)** |  |
| 40 - 60% | 0.04 (-0.02, 0.10) | **0.11 (0.00, 0.21)** | 0.01 (-0.04, 0.06) |
| 20 - 40% | **0.08 (0.01, 0.14)** | **0.19 (0.09, 0.30)** | 0.09 (0.00, 0.19) |
| 0 - 20% | **0.11 (0.04, 0.17)** | **0.13 (0.04, 0.22)** | **0.19 (0.15 0.23)** |
| Intercept | 0.63 (0.57, 0.68) | 0.39 (0.31, 0.48) | 0.52 (0.49, 0.56) |
|  |  |  |  |
| **2: Poverty estimate certainty > 50% in 5 km catchment areas** | β (95% CI) | β (95% CI) | β (95% CI) |
|  | N=483 | N=240 | N=146 |
| Poverty level |  |  |  |
| 80%+ | 0.00 (REF) | 0.00 (REF) | 0.00 (REF) |
| 60 - 80% | **0.06 (0.00, 0.12)** | 0.09 (-0.03, 0.21) |  |
| 40 - 60% | **0.05 (0.02, 0.08)** | **0.14 (0.01, 0.28)** | 0.03 (-0.04, 0.10) |
| 20 - 40% | **0.12 (0.08, 0.16)** | **0.27 (0.13, 0.40)** | **0.12 (0.03, 0.21)** |
| 0 - 20% | **0.16 (0.12, 0.20)** | **0.19 (0.07, 0.31)** | **0.14 (0.01, 0.27)** |
| Intercept | 0.59 (0.57, 0.61) | 0.34 (0.22, 0.46) | 0.50 (0.45, 0.55) |
|  |  |  |  |
| **3: County analysis with DHS wealth quintile** | β (95% CI) | β (95% CI) | β (95% CI) |
|  | N=47 | N=46 | N=42 |
| Wealth quintile |  |  |  |
| Poorest | 0.00 (REF) | 0.00 (REF) | 0.00 (REF) |
| Poorer | 0.05 (-0.04, 0.15) | -0.06 (-0.23, 0.10) | 0.01 (-0.12, 0.14) |
| Middle | 0.06 (-0.02, 0.14) | 0.02 (-0.11, 0.15) | 0.04 (-0.07, 0.15) |
| Richer | **0.14 (0.05, 0.23)** | 0.13 (-0.02, 0.28) | **0.18 (0.06, 0.30)** |
| Richest | **0.14 (0.02, 0.27)** | 0.00 (-0.21, 0.20) | 0.15 (-0.01, 0.30) |
| Intercept | 0.67 (0.60, 0.73) | 0.53 (0.41, 0.64) | 0.51 (0.41, 0.61) |

All models clustered by county to account for correlation between catchment areas of neighboring facilities

Analyses 1 and 2 for clinical quality of delivery care use facilities in areas with 60%+ poverty as the reference category due to the small number of facilities in the poorest group.

Results in bold are significant at p≤0.05.
